# Histone Deacetylase 1 and p300 Can Directly Associate with Chromatin and Compete for Binding in a Mutually Exclusive Manner

**DOI:** 10.1371/journal.pone.0094523

**Published:** 2014-04-10

**Authors:** Xuehui Li, Hui Yang, Suming Huang, Yi Qiu

**Affiliations:** 1 Department of Anatomy and Cell Biology, University of Florida, Gainesville, Florida, United States of America; 2 Department of Biochemistry and Molecular Biology, University of Florida, Gainesville, Florida, United States of America; Texas A&M University, United States of America

## Abstract

Lysine acetyltransferases (KATs) and histone deacetylases (HDACs) are important epigenetic modifiers and dynamically cycled on active gene promoters to regulate transcription. Although HDACs are recruited to gene promoters and DNA hypersensitive sites through interactions with DNA binding factors, HDAC activities are also found globally in intergenic regions where DNA binding factors are not present. It is suggested that HDACs are recruited to those regions through other distinct, yet undefined mechanisms. Here we show that HDACs can be directly recruited to chromatin in the absence of other factors through direct interactions with both DNA and core histone subunits. HDACs interact with DNA in a non-sequence specific manner. HDAC1 and p300 directly bind to the overlapping regions of the histone H3 tail and compete for histone binding. Previously we show that p300 can acetylate HDAC1 to attenuate deacetylase activity. Here we have further mapped two distinct regions of HDAC1 that interact with p300. Interestingly, these regions of HDAC1 also associate with histone H3. More importantly, p300 and HDAC1 compete for chromatin binding both in vitro and in vivo. Therefore, the mutually exclusive associations of HDAC1/p300, p300/histone, and HDAC1/histone on chromatin contribute to the dynamic regulation of histone acetylation by balancing HDAC or KAT activity present at histones to reorganize chromatin structure and regulate transcription.

## Introduction

The reversible acetylation of histones and non-histone proteins by lysine acetyltransferases (KATs) and histone deacetylases (HDACs) plays a critical role in transcriptional regulation and many other cellular processes in eukaryotic cells. Acetylation of histone by KATs commonly correlates with the open chromatin structures required for the binding of multiple transcription factors and leads to transcriptional activation [1]. In contrast, the removal of acetyl groups from histones by HDACs frequently accompanies the suppression of gene activity [2]. The balance of histone acetylation by HDAC and KAT activities is very critical for maintaining unique gene expression patterns for cell growth and development.

Mammalian HDACs are classified into four classes (I, II, III and IV) based on phylogenetic analysis and the sequence homology of the yeast histone deacetylases. Class I HDACs include HDAC1, 2, 3 and 8 (homologous to reduced potassium dependency, Rpd3) and are ubiquitously expressed. Class II HDACs contain HDACs 4, 5, 6, 7, 9 and 10 (homologous to histone deacetylase1, Hda1). In contrast to class I HDACs, class II HDACs are expressed in a more tissue-specific manner. Class III enzymes, including Sirt1, 2, 3, 4, 5, 6, and 7 (homologous to silent information regulator 2, Sir2), require the coenzyme NAD+ as a cofactor. HDAC11 belongs to the class IV family [3]. Although the precise cellular functions of the different HDAC enzymes are poorly understood, evidence suggests that different members of the HDAC family have distinct functions involved in various cellular pathways [4], [5].

The CREB-binding protein (CBP) and p300 are members of the KAT family. p300 and CBP associate with transcription factors and play an essential role in regulating growth and differentiation [6]. p300 interacts with a variety of gene regulators, such as various transcription factors [7], [8], as well as the basal transcription machinery [9]. Interestingly, p300 also interacts with class I HDAC1 and attenuates deacetylase activity through HDAC1 acetylation, indicating a cross-talk between acetyltransferase and deacetylase in regulating a dynamic acetylation status of histones [10], [11].

A dynamic equilibrium between histone acetylation and deacetylation is critical for gene transcription control. The spontaneously increased acetylation of histone in response to deacetylase inhibitor indicates the simultaneous presence of both acetyltransferases and deacetylases at the same gene regulatory loci [12]. Recently, genome-wide mapping analysis found that high levels of HDACs and KATs are recruited to active genes to regulate transcription [13]. The emerging model suggests that KATs and HDACs are constantly cycled on active promoters to promote transcription and restore chromatin integrity after an active round of transcription [14]. Therefore, it is possible that KATs and HDACs are recruited to the chromatin through similar mechanisms. It is generally viewed that KATs and HDACs are recruited to specific locations of chromatin through interacting with DNA binding proteins or protein complexes [15]. However, the presence of HDACs at chromatin regions with no evidence of any other binding factors suggests that HDACs may be recruited to chromatin through other undefined mechanism [16]. It has been shown that p300 can also be recruited to chromatin through direct interaction with histones [17]. Therefore, it became critical to investigate whether HDACs are also directly recruited to chromatins.

In the present study, we show that HDACs can be recruited to chromatin through direct interaction with DNA and all subunits of histones. Furthermore, p300 and HDAC1 compete for chromatin binding by competing with overlapping histone binding sites both in vitro and in vivo. Therefore, dynamic interactions among p300, HDAC1 and histones may play a central role in regulating gene expression.

## Materials and Methods

### Cell culture and transfection

HCT116 colon cancer cell line was cultured in Dulbecco's Modified Eagle Medium (DMEM) supplemented with 10% fetal bovine serum, 100 U/mL of penicillin and 100 μg/mL of streptomycin.

Human p300 shRNA and scramble shRNA were obtained from TRC shRNA library (Open Biosystems, Thermo Fisher Scientific, consortium number # TRCN0000039883). The p300 shRNA or scramble shRNA were cotransfected with psPAX2 packaging plasmid and PMD2.G envelope plasmid into HEK 293FT cells according to the manufacturer's instruction. The culture medium that contained lentiviral particles were collected and added into HCT116 cells in the presence of 8 ug/mL polybrene (Sigma-Aldrich). One day after infection, the cells were selected in DMEM growth medium containing 1 ug/mL puromycin (Sigma-Aldrich) for one week. The plasmids, pGEM-3Z/601, pcDNA3.1-HDAC1, or pcDNA3.1 were transfected into the knockdown cells using Lipofectamine™ 2000 (Invitrogen) according to the manufacturer's instruction.

### Plasmid constructs

HDAC1 deletion mutants were constructed as previously described [18]. HDAC1 H141A and HDAC1 6Q mutant were also constructed as previously described [11]. Histone H3 deletion mutants were generated by PCR and subsequently cloned into the pGEX4T-3 vector (GE healthcare). All constructs were confirmed by DNA sequencing. The plasmids of GST-tagged human histone subunits were kindly provided by Dr. Edward Seto (H. Lee Moffitt Cancer Center, Tampa, FL).

### Protein purification

Flag-tagged HDACs, CoREST, LSD1 and p300 were purified from baculovirus infected insect cells as previously described [11]. Purification of GST-tagged HDAC1 mutants and histone H3 mutants was as described [18]. GST-tagged HDAC1 was eluted by 50 mM Glutathione in 50 μM Tris pH 8.0 buffer. The quality and concentration of the proteins were determined by SDS–PAGE.

### Reconstitution of mononucleosome in vitro

Mononucleosome particles were assembled by salt dilution as described [19] with core histone octamers purified from HeLa cell nuclei [20]. Briefly, 3.75 μg of biotin-labelled 220 bp 601 DNA sequence [21] or MMTV promoter sequence (−105 to +91 bp) was incubated with 4.5 μg of core histones in 50 uL of reconstitution buffer (10 mM Tris pH 7.5, 2 M NaCl, 0.1 mg/mL BSA, 0.5 mM benzamidine). The samples were dialyzed excessively against the reconstitution buffer that contained 1.25, 1.0, and 0.75 M of NaCl, each for 1 h at 4°C. Thereafter, the samples were dialyzed against TE for 1 h at 4°C, before stored at 4°C.

The 100 uL of Dynabeads M-280 Streptavidin (10 mg Dynabeads/mL, ∼6–7X10^8^/mL, Invitrogen) were firstly washed three times with the buffer that contains 5 mM TrisHCl pH 7.5, 0.5 mM EDTA, and 1 M NaCl and resuspended in 100 uL binding buffer (20 mM Tris HCl pH 7.5, 150 mM NaCl, 5 mM MgCl2, 1 mM EDTA, 1 mM EGTA, 5 mM DTT, 1 mg/mL BSA, 10% glycerol, and 1X protease inhibitor cocktail (Roche)) with 0.05% NP-40. The 50 uL of reconstituted biotin labelled mononucleosomes were then added to resuspended beads at 4°C for 2 h with rotation. The bound nucleosomes were washed three times with the binding buffer and resuspended in 150 uL of the binding buffer.

### Mononucleosome pull-down assay

For the pull down assay, 20 uL of immobilized nucleosomes was incubated with 100 ng of Flag-tagged recombinant protein in 100 uL of the binding buffer for 30 min at room temperature with rotation. The incubation was followed by three washes with the binding buffer without BSA. The bound proteins were then separated by SDS–PAGE and detected by Western blotting with an anti-Flag antibody.

For competitive pull-down assay, 20 uL of immobilized mononucleosomes were firstly incubated with 100 ng of either Flag-tagged HDAC1 or p300 in 100 uL of the binding buffer for 20 min at room temperature. Then, 1 or 3 folds of competitor protein was added to the bead mixture and incubated for 20 min at room temperature. The beads were then washed extensively and the level of the bound proteins was examined by Western blotting.

### DNA pull-down assay

The DNA fragments were amplified by PCR with biotin labelled primers. Three hundred ng of purified DNA fragments were immobilized to 5 ul of streptavidin-coupled Dynabeads by incubating with 100 uL of the buffer that contains 5 mM TrisHCl pH 7.5, 0.5 mM EDTA, and 1 M NaCl at room temperature for 15 min. The bound DNA was washed three times with the same buffer.

For DNA pull down assay, 300 ng of bound DNA fragments were pre-incubated with 100 uL of the binding buffer for 15 min at room temperature with rotation. One hundred ng of Flag-tagged proteins or GST-tagged proteins were then added into the immobilized DNA fragments mixture and incubated for additional 30 min at room temperature with rotation. The incubation was followed by three washes with the pull-down buffer without BSA. The bound proteins were then separated by SDS–PAGE and detected by Western blotting with an anti-Flag antibody.

For dIdC competitive pull-down assay, 300 ng of immobilized DNA fragments were incubated with 100 ng of purified Flag-tagged protein in 100 uL of the binding buffer for 20 min at room temperature. Then, the increased amount (2 or 5 folds) of sheared Poly(dI)Poly(dC) double strand (200–300 bp) (Amersham Pharmacia Biotech Inc) was added to the bead mixture and incubated for 20 min at room temperature. The beads were then washed extensively and the level of bound proteins was examined by Western blotting.

### In vitro GST pull-down assay

Bacteria-expressed GST or GST-tagged proteins were immobilized on glutathione-sepharose 4B beads (Pfizer, New York, NY). Three hundred ng of immobilized GST-tagged protein were incubated with 100 ng of purified Flag-tagged proteins in 150 uL of GST binding buffer (150 mM NaCl, 50 mM Tris-HCl, pH 8.0, 2 mM EDTA, 0.1% Nonidet P40 and protease inhibitor cocktail) for 30 min at room temperature. The beads were then washed with the binding buffer and the bound proteins were separated by SDS-PAGE and detected by Western blotting with an anti-Flag antibody.

For GST competition pull-down assay, 300 ng of immobilized GST-tagged histone H3 bound beads were incubated with 100 ng of either Flag-tagged HDAC1 or Flag-tagged p300 in 150 uL of the binding buffer. After 20 min of incubation at room temperature, 1 or 3 folds of competitor protein, or 50 ug or 150 ug of HDAC1 associated complexes extracted from 3134 cells [18] were mixed with immobilized histone H3 in 200 uL of the binding buffer and incubated for another 20 min at room temperature. The beads were then washed extensively and the bound proteins were detected by Western blotting.

### Immunoprecipitation and western blot analysis

The immunoprecipitation and western blot assays were performed as previously described [11] with the following antibodies: anti-Flag (Sigma-Aldrich), anti-HDAC1 (Thermo-Fisher Scientific), anti-p300 (Santa Cruz Biotechnology), anti-H3 (Abcam), and anti-acetyl-H3 (K9K14) (EMD Millipore).

### Chromatin immunoprecipitation (ChIP) and sequential ChIP analysis

ChIP analysis was carried out as described previously [11]. Briefly, the crosslinked cells (5×10^6^ cells per ChIP) were suspended in SDS lysis buffer (50 mM Tris pH 8.0, 10 mM EDTA, 0.25% SDS, Protease inhibitors cocktail) and sonicated for five rounds of five 30-second pulse cycles at maximum power using a Bioruptor (Diagenode Inc) to shear DNA into 200–500 bp of average length. After centrifugation, the samples were precleared with 5 ug/mL rabbit IgG or 50 uL/mL rabbit preimmune serum, and protein A-agarose beads. The precleared chromatin was immunoprecipitated with 5 μg of antibodies. Isolated DNA from immunoprecipitation was subjected for quantitative PCR (qPCR) with primers listed in Supplemental Table 1. Enrichment for a specific DNA sequence was calculated by comparing the amplification value relative to the input.

For sequential ChIP assay, 2×10^7^ HCT116 cells were used for each assay. The first round of ChIP was performed with either anti-p300 antibody or HDAC1 antibody. The precipitated chromatin was washed and eluted as described [22], [23] with minor modifications. Briefly, after elution, the samples were dialyzed with the ChIP buffer until no visible precipitation was observed at 4°C. The elute was diluted to 1 mL by adding 500 uL ChIP buffer and then subjected to a second round of ChIP with anti- histone H3, anti-p300, or anti-HDAC1 antibodies. The samples were then eluted and reversing cross-linked as for standard ChIP assays. Isolated DNA from ChIP was detected by qPCR. Data were calculated by comparing the amplification value relative to the value from the input and then expressed as fold change relative to the value from IgG control. All ChIP experiments were repeated at least three times with independent preparations of cells.

## Results

### HDACs are directly recruited to reconstituted mononucleosomes

Although HDACs are mainly targeted to promoter and hypersensitive sites, the global binding study shows that Rpd3 binds globally at a low level, in addition to binding at a high level at the promoter region [16]. This suggests that HDACs may bind to chromatin independently of sequence-specific DNA-binding proteins for global histone deacetylation [24]. One possible mechanism for this global binding of HDACs is that HDACs can be directly recruited to chromatin by itself.

**Figure 1:**
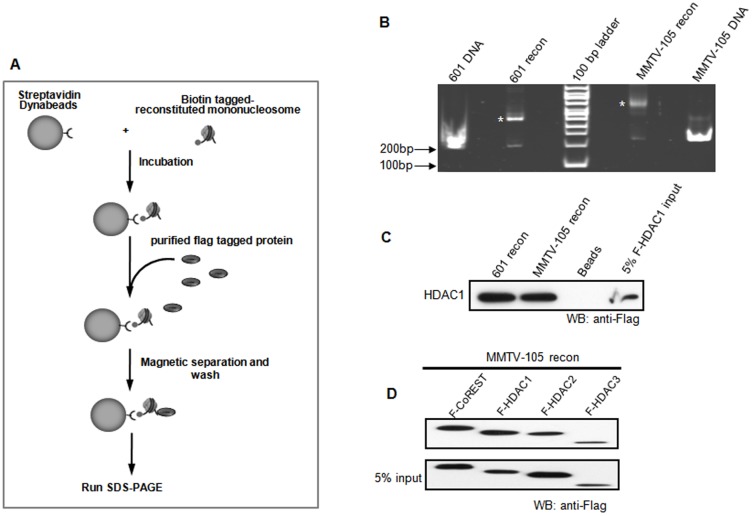
HDACs directly interact with the reconstituted mononucleosomes. (**A**) Schematic representation of mononucleosome pull-down assay using streptavidin-coupled Dynabeads. The biotin labelled monoucleosome was immobilized on streptavidin-bound Dynabeads (Invitrogen). The purified Flag-tagged proteins were incubated with the reconstituted mononucleosome on streptavidin-bound Dynabeads and the bound fraction was subjected to SDS-PAGE and detected by Western blotting. (**B**) Reconstitution of mononucleosomes (recon). Mononuleosome particles were reconstituted using the salt dialysis method with core histones and biotin-labelled DNA fragments. The DNA fragments are 601 DNA, a well characterized non-natural strong nucleosome-positioning sequence [21], and MMTV promoter region. Reconstituted mononucleosomes were then separated on native PAGE and stained by ethidium bromide. * Mononucleosome. (**C**) Recruitment of HDAC1 by 601 recon or MMTV recon. The purified Flag-tagged HDAC1 was incubated with the reconstituted mononucleosome. After extensive washes, proteins bound to the Dynabeads were separated in SDS-PAGE and detected by Western blotting with the anti-Flag antibody. Beads: Flag-tagged HDAC1 was incubated with Dynabeads as a negative control. (**D**) Recruitment of other HDACs by MMTV recon. The purified Flag-tagged HDAC1, 2, 3, and CoREST were incubated with the reconstituted MMTV mononucleosome. After extensive washes, proteins bound to the Dynabeads were separated in SDS-PAGE and detected by Western blotting with the anti-Flag antibody. Each experiment was repeated three times.

To investigate the potential direct HDAC recruitment to chromatin, *in vitro* binding assay was performed to test the associations between HDACs and the reconstituted mononucleosomes. *In vitro* reconstituted biotin labelled mononucleosomes were first coupled onto streptavidin coated magnetic beads, followed by the incubation with Flag-tagged class I deacetylases, which were purified from baculovirus infected insect cells (Figure 1A and Figure S1A in File SI). The purified Flag-HDAC1 did not associate with other potential DNA binding proteins, such as RbAp46 and RbAp48 (Figure S1B in File SI)[25]. Both 601 [21] and mouse mammary tumor virus (MMTV) promoter sequence reconstituted mononucleosomes (Figure 1B) could directly pull down Flag-tagged HDAC1 (Figure 1C and Figure S1C in File SI). The binding affinity of HDAC1 to MMTV-reconstituted mononucleosomes was similar to the binding to 601-reconstituted mononucleosomes. Furthermore, MMTV-reconstituted mononucleosomes also directly interacted with HDAC2 and HDAC3 (Figure 1D). Flag-tagged CoREST, a SANT domain containing protein which can interact with the histone tail and DNA [26], [27], [28], was employed as a positive control (Figure 1D). These data further demonstrate that HDACs can be recruited to chromatin independent of other DNA binding factors.

### HDACs interact with DNA in a non-sequence specific manner

**Figure 2:**
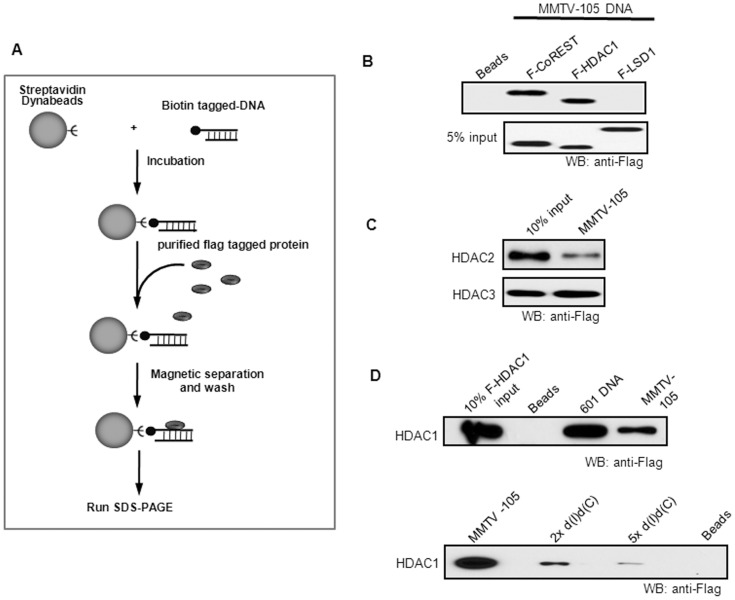
HDACs physically associate with DNA fragments. (**A**) Schematic representation of DNA pull-down assay. The biotin-labelled DNA was immobilized on streptavidin-bound Dynabeads and incubated with Flag-tagged proteins. DNA associated protein was detected by Western blotting. (**B**) and (**C**) Recruitment of HDACs by MMTV promoter sequence. The purified Flag-tagged HDAC1 (**B**) or HDAC2, 3 (**C**), LSD1, and CoREST were incubated with MMTV promoter DNA fragments. Proteins bound to DNA were separated in SDS-PAGE and detected by Western blotting. Beads: Flag-tagged HDAC1, HDAC2, and LSD1 were incubated with Dynabeads as a negative control. (**D**) Non-sequence-specific binding of HDAC1 to DNA. The purified Flag-tagged HDAC1 was incubated with 601 DNA fragments, or with MMTV promoter DNA fragments in the presence of dIdC. Beads: Flag-tagged HDAC1 was incubated with Dynabeads as a negative control. The proteins associated with DNA were detected by Western blotting. Each experiment was repeated at least three times.

Next, we investigated whether HDACs interact with DNA. The biotin labelled DNA fragments were coupled onto streptavidin conjugated magnetic beads, followed by incubation with Flag-tagged HDACs (Figure 2A). MMTV promoter DNA interacted with HDAC1. The binding of Flag-tagged CoREST and LSD1 to DNA was also tested as positive and negative controls (Figure 2B). It has been shown that LSD1 does not bind to DNA [27] while CoREST does [28]. HDAC2 and HDAC3 also interacted with the MMTV DNA sequence directly (Figure 2C and Figure S1D in File SI). To examine whether this interaction is sequence-specific, 601 DNA fragment, a sequence-unspecific DNA, was incubated with HDAC1. 601 DNA also had a strong interaction with HDAC1 (Figure 2D), suggesting a non-sequence specific association between HDAC1 and DNA. To further test the DNA sequence specificity, a competition assay was performed. The increased amount of sheared d(I)d(C) was added with MMTV promoter DNA and HDAC1. The d(I)d(C) competed with MMTV for the binding of HDAC1, demonstrating that the interaction between HDAC1 and DNA is non-sequence specific (Figure 2D). To further confirm the direct binding of HDAC1 to DNA, we used GST-HDAC1 purified from bacteria to incubate with DNA. HDAC1 interacted with different DNA fragments (Figures S2C and S2D in File SI). Other DNA fragments from various gene loci were also tested for the binding specificity of HDAC1and HDAC2. Although the interaction with DNA appears to be non-sequence specific, some sequences may be more preferred than others (Figure S2B in File SI). Thus, we conclude that class I HDACs can directly interact with DNA, but in a non-sequence specific manner.

### HDACs interact with all core histone subunits

**Figure 3:**
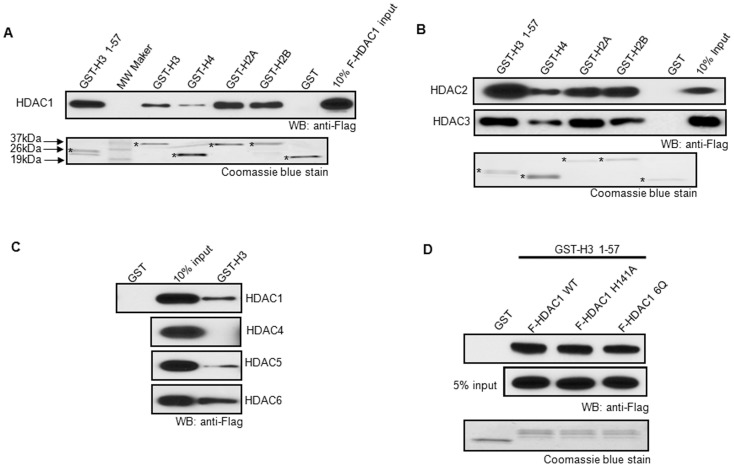
HDACs directly interact with all core histone subunits. (**A**) All histone subunits interact with HDAC1. Flag-tagged HDAC1 was incubated with the purified glutathione sepharose beads immobilized GST-H3, H4, H2A, or H2B in a pull-down assay and histone associated HDAC1 was detected by Western blotting. * indicates the protein of interest in Coomassie blue staining. (**B**) Histone subunits interact with other Class I HDACs. Flag-tagged HDAC 2 and 3 were incubated with the purified GST-histone H3, H4, H2A, or H2B and detected by Western blotting. * indicates the protein of interest in Coomassie blue staining. (**C**) Histone H3 interacts with Class II HDACs. The purified GST-histone H3 was incubated with Flag-tagged HDAC4, 5 and 6. Proteins bound to GST-H3 were separated in SDS-PAGE and detected by Western blotting. (**D**) Histone H3 tail 1-57 interacts with HDAC1 regardless of HDAC1 acetylation. Flag-tagged HDAC1 acetylation mimic mutant 6Q, single mutation of HDAC1 H141A and wild type HDAC1 were incubated with the purified GST-histone H3 1-57. Associated proteins were detected by Western blotting. Each experiment was repeated at least three times.

We next examined whether HDACs physically associate with core histones. Bacterially synthesized GST-histone subunits H3, H4, H2A, and H2B fusion proteins were immobilized in glutathione agarose beads and incubated with Flag-HDAC1. Except histone H4, HDAC1 interacted with all subunits of core histone in a similar affinity (Figure 3A). HDAC2 and 3 also interacted with all subunits of histones (Figure 3B). We also tested class II HDACs, such as HDAC4, HDAC5 and HDAC6. These HDACs can shuttle in and out of nuclear and are important for gene transcription [13], [29], [30]. The result showed that HDAC5 and HDAC6, but not HDAC4, interacted with GST-histone H3 (Figure 3C). Notably, the histone H3 tail (1–57 amino acids) contained complete binding capacity to HDACs (Figures 3A and 3B, and Figure S3 in File SI).

Next, we tested whether HDAC1 modification and deacetylase activity affect the binding to histone. It is known that HDAC1 can be acetylated by p300. Moreover, acetylation on HDAC1 inhibits its deacetylase activity [11]. Therefore, it will be interesting to test whether acetylated HDAC1 can still be recruited to histones. As shown in Figure 3D, HDAC1 mutant 6Q (mimic acetylated HDAC1) interacted with histone H3 tail just as the wild type HDAC1. The single mutation of HDAC1 H141A, which abolished the deacetylase activity, also interacted with the histone H3 tail (Figure 3D). In addition, the treatment with histone deacetylase inhibitor, TSA, did not affect the binding activity of HDAC1 to histone H3 (Figure S4 in Flie SI). Therefore, the interaction between HDAC1 and histone H3 is independent of HDAC1 acetylation and HDAC1 deacetylase activity.

### HDAC1 and p300 interact with core histones regardless of the histone acetylation status

**Figure 4:**
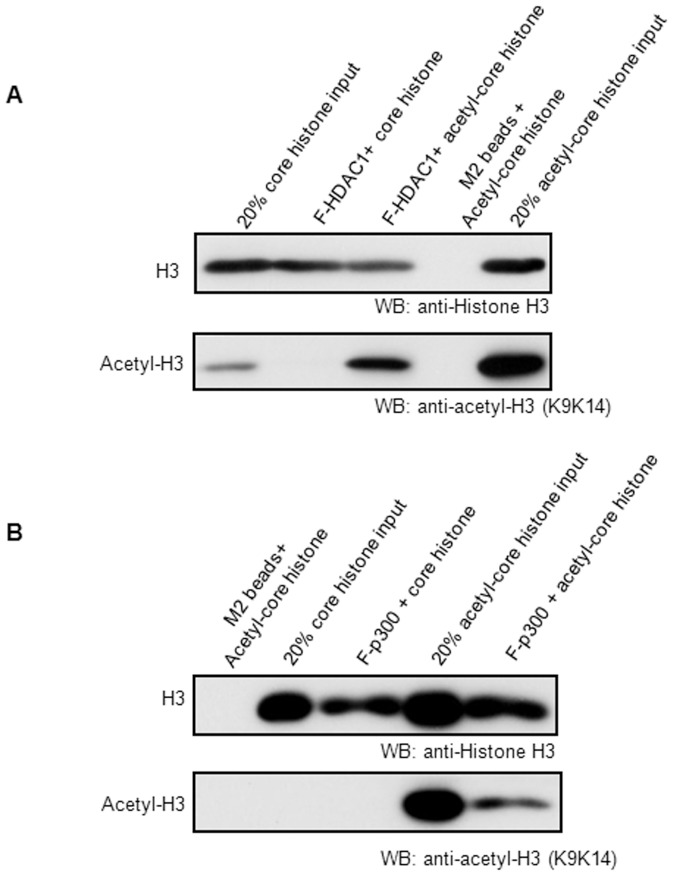
HDACs and p300 directly interact with core histones regardless of the acetylation level. Flag-tagged HDAC1 and p300 were immobilized on M2 beads. The purified hyper-acetylated or untreated HeLa core histones were incubated with immobilized HDAC1 (**A**) and p300 (**B**). Bound histones were detected by Western blotting with the anti-histone H3 and anti-acetyl histone H3 antibodies. The experiments were repeated three times.

We further examined whether the acetylation status of histone affects the interaction between HDAC1 and core histones. The core histone particles were isolated from HeLa cells with or without treatment of histone deacetylase inhibitor, sodium butyrate. The isolated hyper-acetylated or normal control core histone particles were then incubated with Flag-tagged HDAC1 immobilized with M2 agarose affinity gel. As shown in Figure 4A, HDAC1 pulled down both hyper-acetylated and normal control core histone particles. Our data here also showed that p300 directly interacted with hyper-acetylated or normal core histones (Figure 4B), which is consistent with the previous publications [17], [31]. These data suggest that HDAC1 and p300 can both be recruited to chromatin in the absence of other DNA or histone binding factors, regardless of the acetylation status of the core histones.

### Histone H3 physically associates with both HDAC1 and p300 through overlapping regions of histone H3 tail

**Figure 5:**
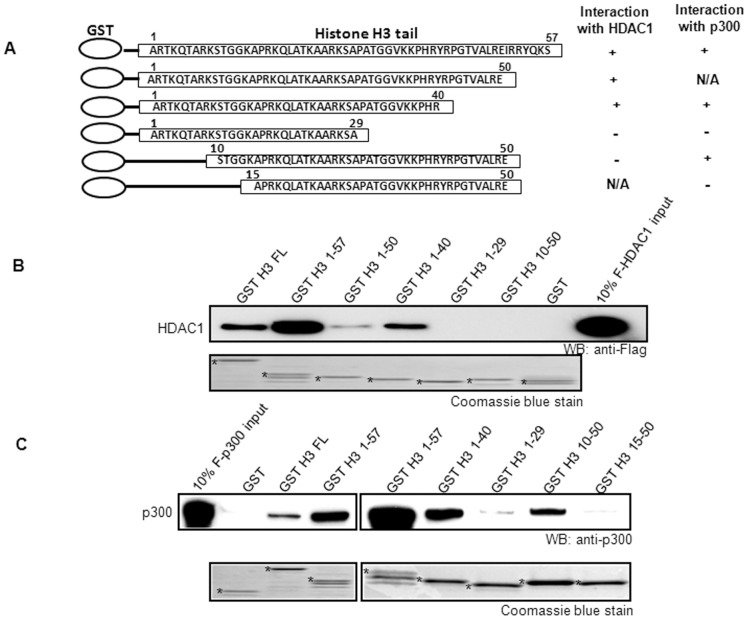
HDAC1 and p300 are recruited by the C-terminal tail of histone H3. (**A**) Schematic representation of the GST-H3 mutant constructs. B and C. Glutathione sepharose beads immobilized GST H3 proteins were incubated with Flag-tagged HDAC1 (**B**) and p300 (**C**) and bound fractions were detected by Western blotting. The experiments were repeated three times.

To further define the binding domains involved in the interactions of HDAC1 and p300 with histone H3, we generated a series of GST-tagged histone H3 tail constructs (Figure 5A). The glutathione sepharose beads immobilized with GST-histone H3 fusion proteins were incubated with Flag-tagged HDAC1 in order to identify the interacting region of histone H3. The N-terminal region (1–40aa) of histone H3 significantly interacted with HDAC1 (Figure 5B). The histone H3 interacting domain with p300 was also examined. Interestingly, p300 also interacted with the 1–40aa region of histone H3 (Figure 5C). However, unlike HDAC1, p300 interacted with the 10–50aa region of histone H3 as well, suggests that p300 and HDAC1 likely bind to overlapping but distinct regions of histone H3.

### HDAC1 interacts with the core histones through two distinct regions

**Figure 6:**
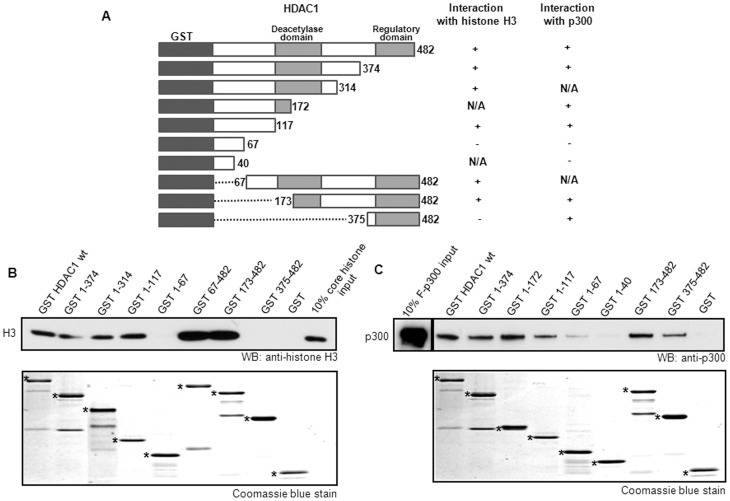
HDAC1 interacts with core histones p300 through two distinct regions. (**A**) Schematic representation of the GST-HDAC1 mutant constructs used in GST pull-down assay with core histones and p300. Glutathione sepharose beads immobilized GST HDAC1 proteins were incubated with core histones (**B**) and Flag-tagged p300 (**C**). The bound fractions were detected by Western blotting. The experiments were repeated three times.

Next, we investigated the HDAC1 domain that interacts with the core histones. A series of GST-HDAC1 fusion proteins containing distinct regions of HDAC1 [18] were purified and incubated with purified core histones (Figure 6A). The N-terminal region of HDAC1 1–117aa was found to interact with histone (Figure 6B). In addition, a region between 173 to 482aa also interacted with core histones.

### HDAC1 interacts with p300 through two independent domains

It has been shown that HDAC1 interacts with the C/H3 domain of p300 [10]. However, the interaction domain on HDAC1 has not yet been defined. To identify the binding domains of HDAC1 involved in such interaction, GST-HDAC1 fusion proteins were incubated with p300 (Figure 6A). Two distinct regions of HDAC1 interacted with p300, the N-terminal region of HDAC1 1–117aa which also interacted with histone, and the C-terminal region (HDAC1 375-482aa) (Figure 6C). Therefore, two binding domains of HDAC1 are independently involved in the interaction with p300.

### HDAC1 and its associated corepressor complexes compete with p300 for histone binding

**Figure 7:**
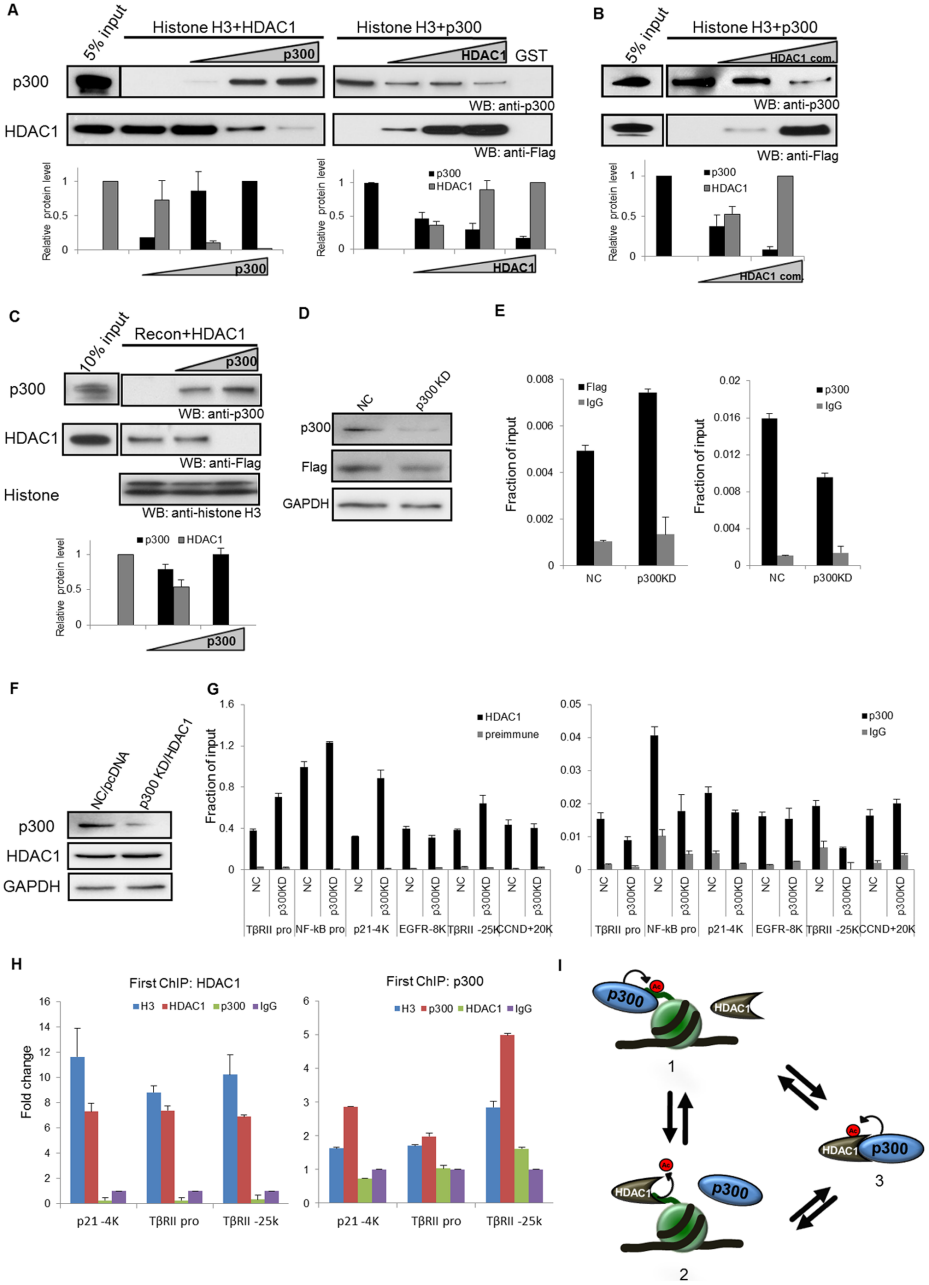
HDAC1 and p300 are directly recruited to the nucleosome in a competitive manner. (A) HDAC1 and p300 competitively interact with GST-histone H3. Glutathione sepharose beads immobilized GST histone H3 was first incubated with HDAC1 and subsequently incubated with various concentrations of p300 and vice versa. The GST-histone H3 bound p300 and HDAC1 were detected by Western blotting with anti-Flag and anti-p300 antibodies. (**B**) HDAC1 associated complexes and p300 competitively interacts with GST-histone H3. Flag tagged HDAC1 associated corepressor complexes were purified from 3134 cells [18]. Immobilized GST histone H3 was first incubated with p300 and subsequently incubated with various concentrations of HDAC1 complexes. The GST-histone H3 bound p300 and HDAC1 were detected by Western blotting with anti-Flag and anti-p300 antibodies. The experiment was repeated twice. (**C**) HDAC1 and p300 competitively interact with reconstituted mononuleosomes. The reconstituted mononuleosomes were first immobilized on streptavidin coated magnetic beads and incubated with Flag-HDAC1; then, the increased amount of p300 was added to the mixture. After extensive washes, the proteins that remained on the beads were separated by SDS-PAGE and detected by Western blotting with anti-HDAC1 and anti-p300 antibodies. Quantifications of band density (A-C) are shown in the histograms. Gel-pro analyser was used to analyse the intensity of each band. Error bars represent standard deviation (n = 3). (**D**) Flag-HDAC1 and 601 plasmid are cotransfected in p300 knockdown (p300 KD) and control HCT116 cells (NC). The protein levels were detected by Western blotting with anti-p300, anti-Flag, and anti-GAPDH antibodies. (**E**) Flag-HDAC1 binding at 601 positioned nucleosome is increased in p300 knockdown cells. 601 and Flag-HDAC1 were cotransfected into p300 knockdown or scramble control HCT116 cells. Anti-Flag and anti-p300 antibodies were used to perform HDAC1 and p300 CHIP analysis. Rabbit IgG was used as a negative control. (**F**) The HDAC1 protein level is normalized by transfecting small amount of HDAC1 in p300 knockdown cells. The protein levels in HDAC1 transfected p300 knockdown cells (p300 KD/HDAC1) and control cells (NC/pcDNA) were examined by Western blotting with anti-p300, anti-HDAC1, and anti-GAPDH antibodies. (**G**) Dynamic HDAC1 and p300 binding at diverse gene loci in p300 knockdown HCT116 cells. Anti-p300 and anti-HDAC1 specific antibodies were used to perform HDAC1 and p300 CHIP analysis. Preimmune serum and rabbit IgG were used as negative controls. The experiments were repeated three times. (**H**) Sequential ChIP analysis of HDAC1 and p300 co-occupancy at diverse gene loci in HCT116 cells. Left panel: The chromatin was first ChIPed with HDAC1 antibody and subsequently re-ChIPed with indicated antibodies. Right panel: The chromatin was first ChIPed with p300 antibody and subsequently re-ChIPed with indicated antibodies. The fold change is expressed as comparing amplification values to IgG control. (**I**) Schematic representation of the mutually exclusive associations of p300/chromatin, HDAC1/chromatin and p300/HDAC1. 1. p300 associates with chromatin and acetylates chromatin. 2. HDAC1 associates with chromatin and deacetylates chromatin. 3. p300 associates with HDAC1 and acetylates HDAC1. The acetylation attenuates HDAC1 deacetylase activity.

Our data show that p300 and HDAC1 bind to the overlapping region of histone H3, and p300 and histone H3 bind to the overlapping region of HDAC1. We then further investigated whether p300 and HDAC1 compete for histone tail binding sites. Flag-tagged p300 was added to pre-incubated mixtures of HDAC1 and glutathione immobilized GST-histone H3. The increased amount of p300 competed with HDAC1 to bind to GST-histone H3 as shown in Figure 7A. In contrast, the presence of the increased HDAC1 reversed the binding of p300/histone H3 to HDAC1/histone H3. Similarly competitive bindings among HDAC2, p300 and histone H3 were also observed (Figure S5 in File SI).

Since HDAC1 often associates with corepressor complexes in cells, we tested whether HDAC1 complexes can also compete with p300 for histone tail binding. Flag-tagged HDAC1 was stably overexpressed in 3134 cells. The nuclear extract was immunoprecipitated with Flag antibody and eluted with Flag peptide. The complexes contained the mix of Sin3, NuRD and CoREST complexes [18]. The increased amount of HDAC1 complexes competed with p300 to bind to GST-histone H3 (Figure 7B).

### HDAC1 and p300 compete for chromatin binding in vitro and in vivo

We further tested whether this competitive interaction also occurs on chromatin. The mononucleosomes that was immobilized by streptavidin coated magnetic beads were first incubated with HDAC1. With the addition of increased amount of p300, the interaction between HDAC1 and the reconstituted mononucleosomes decreased and the interaction between p300 and mononucleosomes increased (Figure 7C). Next, we wanted to examine whether this competition also occurs in vivo. If this is the case, then knockdown of p300 in cells will enhance HDAC1 binding at the same binding site. We created a stable p300 knockdown HCT116 cell line. Flag-HDAC1 with the 601 plasmid was transfected into the knockdown and control cells to examine HDAC1 recruitment (Figure 7D). Flag-HDAC1 binding to 601 nucleosome increased in p300 knockdown cells compared to the control cells, while the recruitment of p300 decreased in p300 knockdown cells (Figure 7E). These data support the competitive binding to chromatin between p300 and HDAC1. The result led us to further investigate whether the competitive interaction occurs at an endogenous gene locus. In order to test that, we examined several targeted gene loci in which both p300 and HDAC1 were recruited. The examined binding sites include both promoter and enhancer regions. These regions include promoter regions of TGFβRII and NF-kB, which are also highly occupied by other cofactors; the upstream enhancer regions, such as p21 upstream −4K region and EGFR upstream −8K region, which have low occupancy of other cofactors. We also analysed several intergenic regions, including TGFβRII upstream −25K and CCDN1 downstream +20K regions, which have not been shown to interact with factors other than p300 and HDAC1 [13], [32], [33]. Due to decreased expression of HDAC1 in p300 knockdown HCT116 cells (Figure S6 in File SI), we transfected small amount of HDAC1 plasmid into p300 knockdown cells in order to maintain similar HDAC1 protein level in both cells (Figure 7F). HDAC1 recruitment in p300 knockdown cells increased at some gene loci, such as TGFβRII promoter, NF-kB promoter, p21-4K, and TGFβRII-25K, whereas p300 enrichment was reduced at these loci accordingly (Figure 7G). It is noticed that at some gene loci, such as CCDN +20K and EGFR −8K, HDAC1 recruitment was not increased in p300 knockdown cells, presumably because p300 recruitment at these loci was not reduced in p300 knockdown cells (Figure 7G). To further confirm the competitive binding of HDAC1 and p300, a sequential ChIP was also performed at the same gene loci in HCT116 cells. No co-occupancy of HDAC1 and p300 was observed on these loci (Figure 7H). This is consistent with the result obtained from p300 knockdown cells (Figure 7G). These results demonstrate that HDAC1 and p300 can compete for chromatin binding at endogenous gene locus. Therefore, our data support that p300 and HDAC1 can dynamically interact with chromatin in a mutually exclusive manner both in vitro and in vivo (Figure 7I).

## Discussion

It has been generally reviewed that HDACs are recruited to specific locations of genes by interacting with other DNA binding proteins or complexes. Here we show that HDACs can directly interact with chromatin without an interaction with other chromatin binding factors. HDACs interact with chromatin through DNA binding activity and direct interaction with histones. Although the interaction with DNA appears to be non-sequence specific, some sequences may be more preferred than others (Figure S2 in File SI). However, to understand the functional DNA binding property, the structural analysis of HDACs need to be further developed. p300 may also interact with histone and DNA through a similar manner[17], [34]. The recruitment of HDACs and KATs is likely independent of the acetylation status of histones[17] [31], however, recent study suggests that phosphorylation of serine 10 at histone H3 N-terminal tail blocks the access of HDAC1 and HDAC2 to histone H3 [35].

According to the genome-wide mapping of HDACs and KATs in yeast [16], [36] and humans [13], both HDACs and KATs are highly enriched at promoter regions, where the basal transcription machinery and other DNA binding cofactors are located. HDACs and KATs binding may also be targeted to other chromatin regions, such as enhancer regions and gene coding regions. The recruitment of HDACs and KATs to these regions is often achieved through interaction with variety of factors, such as transcription factors, chromatin interacting factors, Pol II and its associated complexes, and histone modification signals, i.e., H3K36me3 [13], [37], [38], [39], [40], [41], [42], [43], [44]. However, globally, an average of 2 to 2.5 fold enrichment of Rpd3 binding is observed, in addition to the higher magnitude of enrichment at the promoter in yeast [16]. This low level of binding of Rpd3 appears to be recruited throughout the genome in an untargeted manner [45], [46]. The widespread functions of HDACs and KATs appear to occur independently of apparent sequence-specific DNA-binding proteins as 'global histone acetylation and deacetylation' [24]; thus raising a question of how HDACs can be recruited to the genome where there is a lack of binding of transcription factors or other chromatin binding proteins. Our study provides evidence that HDACs can be directly recruited to chromatin. It is possible that the direct recruitment of KATs and HDACs contributes to a globally low level of binding and the interactions with other transcription regulators may strengthen the binding at promoter and hypersensitive site areas.

Co-repressor complex activity is originally considered to associate with repressed genes and is replaced by co activators during gene activation. Recent advancements of technologies have allowed for the study of gene regulation on a genome-wide scale and have challenged our understanding of co-repressor actions, as HDACs and KATs are mostly recruited to actively transcribed genes [13]. Current models of co-repressor function appreciate the dynamics of the opposing co-activator and co-repressor complexes, a continuous exchange between co-repressor and co-activator complexes [13], [14], [15], [39], [47], [48], [49]. The dynamic replacement theory is also supported by the notion that the bindings of transcription factors, cofactors and HDACs are transient and dynamic at chromatin [50], [51], [52]. However, how the exchange of KATs or HDACs occurs at chromatin is not explained. Mapping of the binding regions for HDAC1 as well as p300 to histone H3 indicates that HDAC1 and p300 share the overlapping interaction region of histone H3. The competition assay shows that the increased association of HDAC1 complexes to histone H3 weakens the binding of p300 to histone H3, or vice versa, which supports the notion that the interaction of histone with HDAC1 or p300 is in an exclusively competitive manner. The in vivo study also indicated that reduced occupancy of p300 on the chromatin enhanced HDAC1 binding both transfected 601 positioned nucleosome and endogenous gene loci. In addition, sequential ChIP analysis further confirms that HDAC1 and p300 do not co-occupy the same gene locus in vivo. This competitive interaction of HDAC1 and p300 to chromatin is likely independent from the binding of other cofactors, since the presence of cofactors at different gene loci do not affect recruitment of HDAC1 to chromatin in p300 knockdown cells. Therefore, the dynamic balance of KAT and HDAC1 on chromatin may be a general mechanism to maintain proper acetylation level of the chromatin.

It is reported that p300 can directly recruit HDAC1 through its C/H3 domain, which suggests another layer of regulation in controlling histone acetylation/deacetylation [10]. The associations between HDACs and KATs have been shown in other studies as well, such as pCAF/HDAC1 [53] and p300/HDAC6 [54]. We further defined two independent regions in HDAC1 to bind to p300. These two regions also mediate the binding of histone H3; therefore, prevent simultaneous binding of p300 and histone H3 to HDAC1. Interestingly, p300 can acetylate HDAC1 and attenuate the deacetylase activity of HDAC1 [11], [18]. Thus, rapid cycling between p300/chromatin, HDAC1/chromatin and p300/HDAC1 contributes to the dynamic regulation of histone acetylation not only by balancing the level of KAT and HDAC recruitment to histone, but also by neutralizing HDAC or KAT activity presented at histone to reorganize the chromatin structure and regulate transcription (Figure 7I).

## Supporting Information

File S1 Figure S1 Direct recruitment of HDACs by MMTV promoter. (A) Recombinant Flag-HDAC1 was expressed from baculovirus infected insect cells and purified through affinity purification. The puified Flag-HDAC1 was subjected to SDS-PAGE and coomassie blue staining. * indicates Flag-HDAC1. The bovine serum albumin (BSA) is served as loading control. (B) Purified Flag-HDAC1 from insect cells and HDAC1 complex (HDAC1 com.) from 3134 cells were subjected to Western blot with HDAC1 and RbAp 46/48 antibodies. #1 and #2 are two biological repeats of purification. (C) Recruitment of HDAC1 by MMTV recon. The purified Flag-tagged HDAC1, CoREST, or LSD1 was incubated with the reconstituted MMTV mononucleosome. After extensive washes, proteins bound to the Dynabeads were separated in SDS-PAGE and detected by Western blotting with the anti-Flag antibody. (D) Recruitment of HDACs by MMTV promoter sequence. The purified Flag-tagged HDAC1, HDAC2, HDAC3, or LSD1 was incubated with MMTV promoter DNA fragments. Proteins bound to DNA were separated in SDS-PAGE and detected by Western blotting. Flag-tagged HDAC1, HDAC2, and HDAC3 were incubated with unbound beads (beads) as a negative control. Figure S2 HDAC1 interacts with various DNA sequences. (A) Schematic representation of biotin labelled DNA fragments. (B) DNA associated Flag tagged HDAC1 and HDAC2 were detected by Western blotting with the anti-Flag antibody. The experiment was repeated three times. (C) Bacterial expressed GST-HDAC1 was purified and eluted by 50 mM Glutathione and incubated with various biotin labelled DNA fragments. DNA associated GST HDAC1 was detected by Western blotting with the anti-HDAC1 antibody. The experiment was repeated three times. (D) Coomassie blue staining of purified GST tagged HDAC1. * indicates GST-HDAC1. Figure S3 Histone H3 tail interacts with HDACs. The purified GST-histone H3 tail 1-57 was incubated with Flag-tagged HDAC1, 2, 3, 4, 5 and 6. Proteins bound to GST-H3 1-57 were separated in SDS-PAGE and detected by Western blotting. The experiment was repeated at least three times. Figure S4 Treatment of TSA does not affect binding of histone H3 with HDAC1. The glutathione sepharose immobilized GST-histone H3 protein was incubated with recombinant flag tagged HDAC1 with or without the presence of 100 nM or 300 nM TSA in a pull down assay. Histone H3 associated HDAC1 was detected by Western blotting with an anti-Flag antibody. The experiment was repeated at least three times. Figure S5 HDAC2 and p300 are directly recruited to histone H3 in a competitive manner. The glutathione sepharose immobilized GST-histone H3 protein was first incubated with HDAC2 and subsequently incubated with various concentrations of p300 or vice versa. The histone H3 bound p300 and HDAC2 were detected by Western blotting with anti-Flag and anti-p300 antibodies. The experiment was repeated three times. Figure S6 HDAC1 protein level is decreased in p300 knockdown cells. The protein levels in p300 knockdown cells (p300 KD) and control cells (NC) were examined by Western blotting with anti-p300, anti-HDAC1, and anti-β-actin antibodies. The experiment was repeated three times. Table S1 Primers used in CHIP analysis.
(PPT)
