# Supplementary material for: Histone Deacetylase 1 and p300 Can Directly Associate with Chromatin and Compete for Binding in a Mutually Exclusive Manner
Source: PLoS One. 2014 Apr 10;9(4):e94523. doi: 10.1371/journal.pone.0094523 (PMC3983199; doi:10.1371/journal.pone.0094523)
Supplement: File S1 — Figure S1 Direct recruitment of HDACs by MMTV promoter. (A) Recombinant Flag-HDAC1 was expressed from baculovirus infected insect cells and purified through affinity purification. The puified Flag-HDAC1 was subjected to SDS-PAGE and coomassie blue staining. * indicates Flag-HDAC1. The bovine serum albumin (BSA) is served as loading control. (B) Purified Flag-HDAC1 from insect cells and HDAC1 complex (HDAC1 com.) from 3134 cells were subjected to Western blot with HDAC1 and RbAp 46/48 antibodies. #1 and #2 are two biological repeats of purification. (C) Recruitment of HDAC1 by MMTV recon. The purified Flag-tagged HDAC1, CoREST, or LSD1 was incubated with the reconstituted MMTV mononucleosome. After extensive washes, proteins bound to the Dynabeads were separated in SDS-PAGE and detected by Western blotting with the anti-Flag antibody. (D) Recruitment of HDACs by MMTV promoter sequence. The purified Flag-tagged HDAC1, HDAC2, HDAC3, or LSD1 was incubated with MMTV promoter DNA fragments. Proteins bound to DNA were separated in SDS-PAGE and detected by Western blotting. Flag-tagged HDAC1, HDAC2, and HDAC3 were incubated with unbound beads (beads) as a negative control. Figure S2 HDAC1 interacts with various DNA sequences. (A) Schematic representation of biotin labelled DNA fragments. (B) DNA associated Flag tagged HDAC1 and HDAC2 were detected by Western blotting with the anti-Flag antibody. The experiment was repeated three times. (C) Bacterial expressed GST-HDAC1 was purified and eluted by 50 mM Glutathione and incubated with various biotin labelled DNA fragments. DNA associated GST HDAC1 was detected by Western blotting with the anti-HDAC1 antibody. The experiment was repeated three times. (D) Coomassie blue staining of purified GST tagged HDAC1. * indicates GST-HDAC1. Figure S3 Histone H3 tail interacts with HDACs. The purified GST-histone H3 tail 1-57 was incubated with Flag-tagged HDAC1, 2, 3, 4, 5 and 6. Proteins bound to GST-H3 1-57 were separated in SDS-PAGE an [file pone.0094523.s001.ppt]

## Slide 1
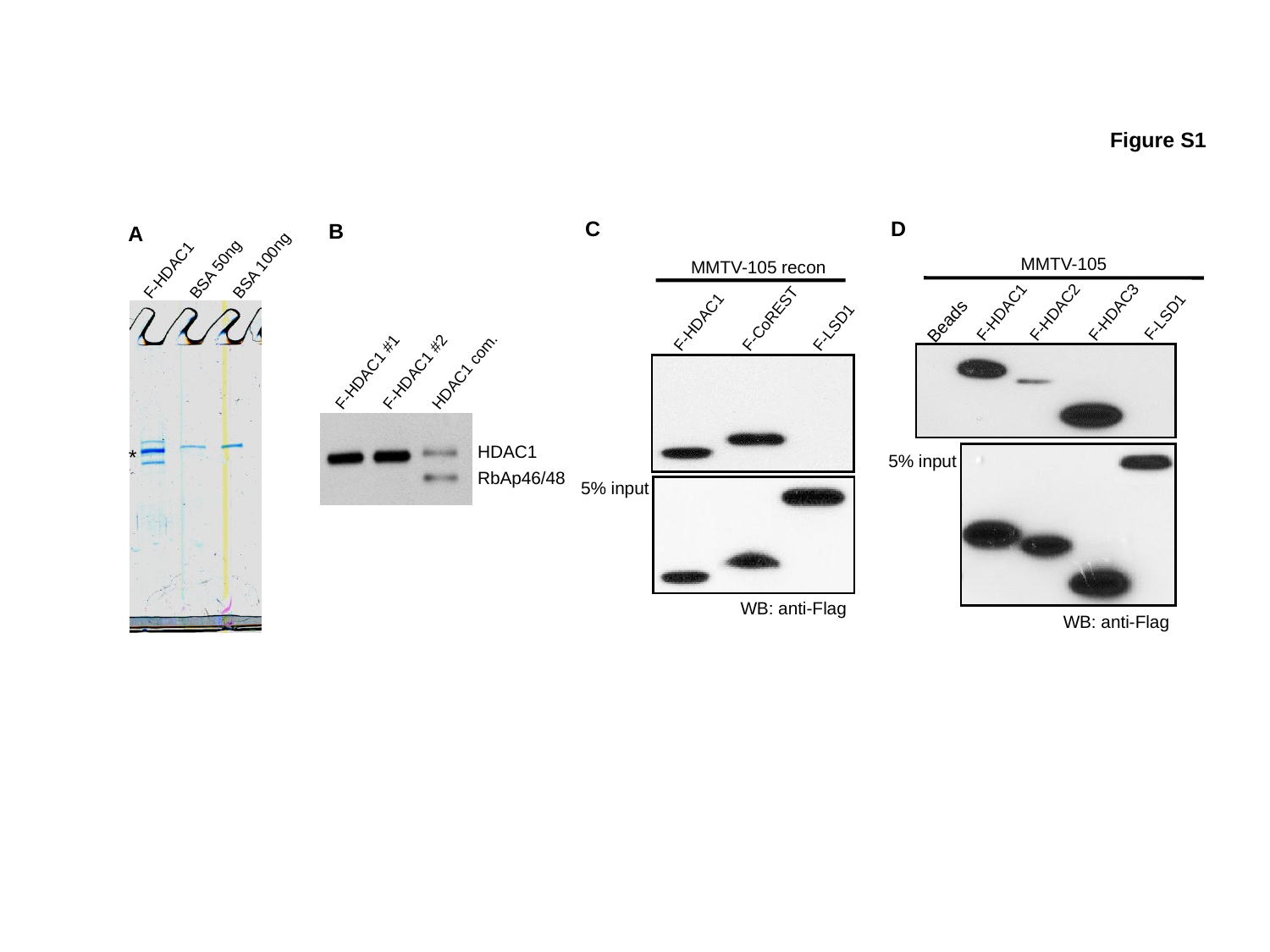

Figure S1
D
C
B
A
MMTV-105
F-HDAC1
F-HDAC2
F-HDAC3
F-LSD1
Beads
BSA 100ng
MMTV-105 recon
BSA 50ng
F-HDAC1
F-CoREST
F-HDAC1
F-LSD1
HDAC1 com.
F-HDAC1 #2
F-HDAC1 #1
HDAC1
*
5% input
RbAp46/48
5% input
WB: anti-Flag
WB: anti-Flag

## Slide 2
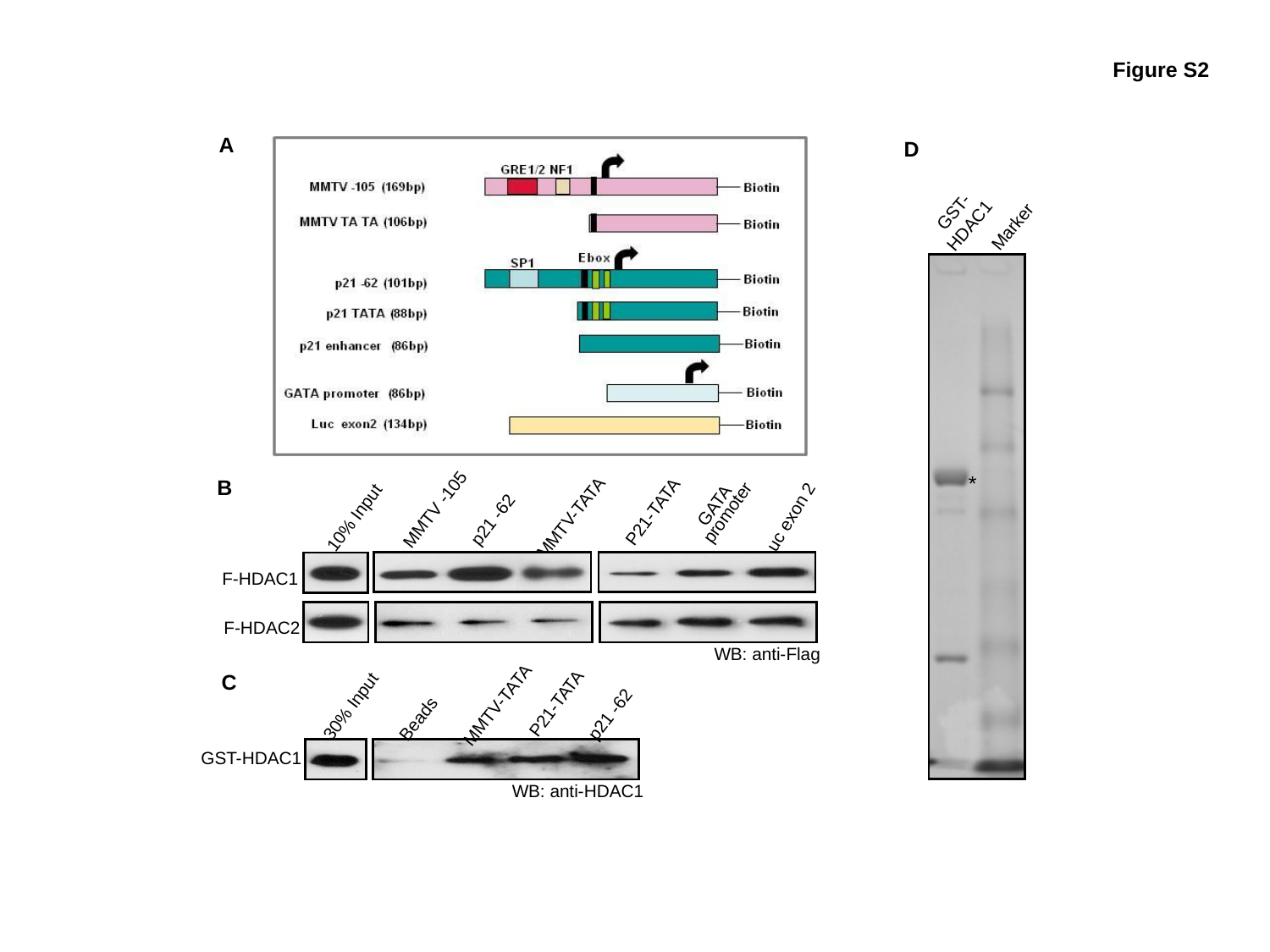

Figure S2
A
D
GST-HDAC1
Marker
P21-TATA
MMTV -105
GATA
promoter
MMTV-TATA
p21 -62
10% Input
F-HDAC1
F-HDAC2
Luc exon 2
B
*
WB: anti-Flag
P21-TATA
MMTV-TATA
30% Input
p21 -62
Beads
C
GST-HDAC1
WB: anti-HDAC1

## Slide 3
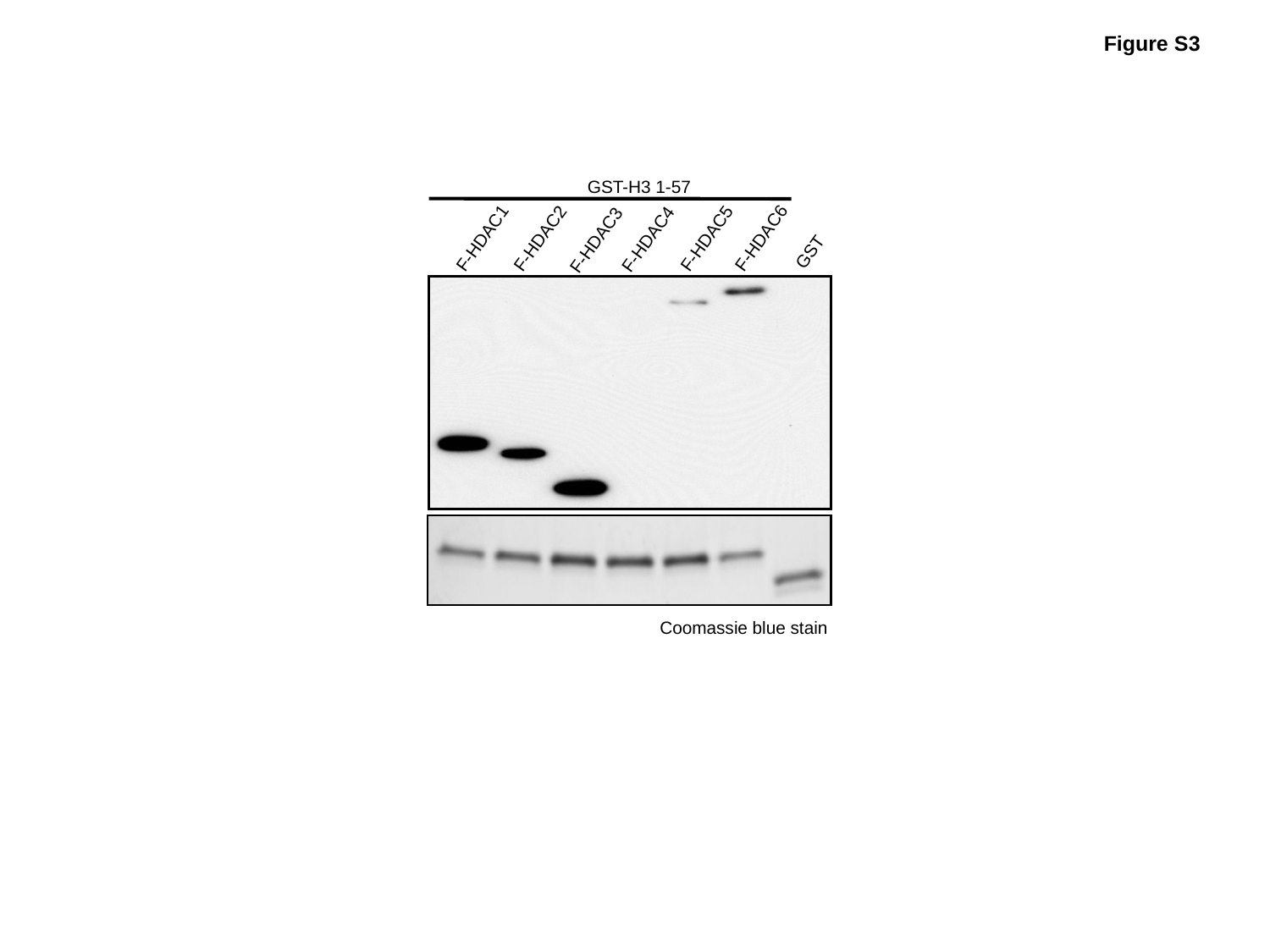

Figure S3
GST-H3 1-57
F-HDAC6
F-HDAC5
F-HDAC1
F-HDAC3
F-HDAC2
F-HDAC4
GST
Coomassie blue stain

## Slide 4
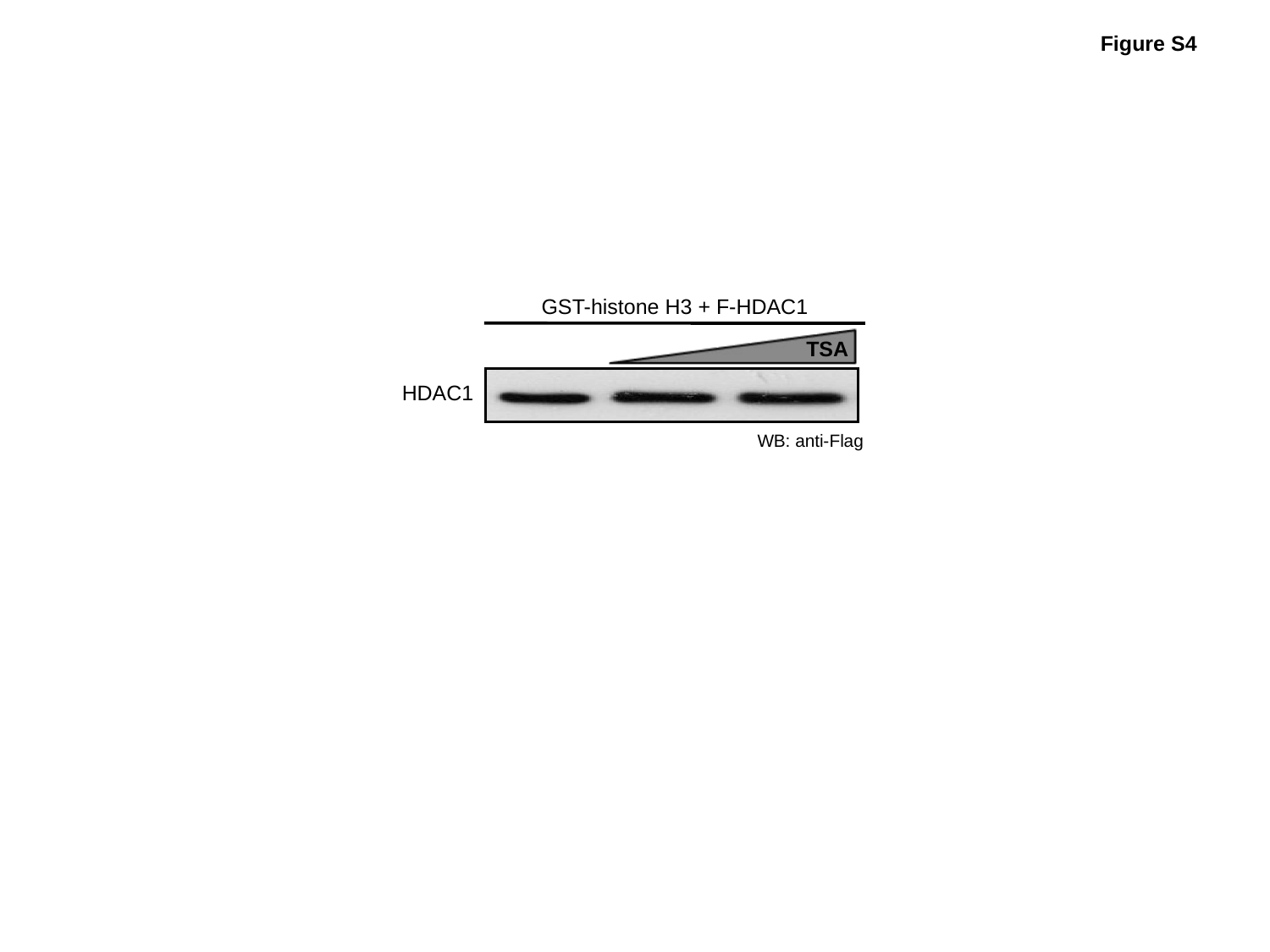

Figure S4
GST-histone H3 + F-HDAC1
TSA
HDAC1
WB: anti-Flag

## Slide 5
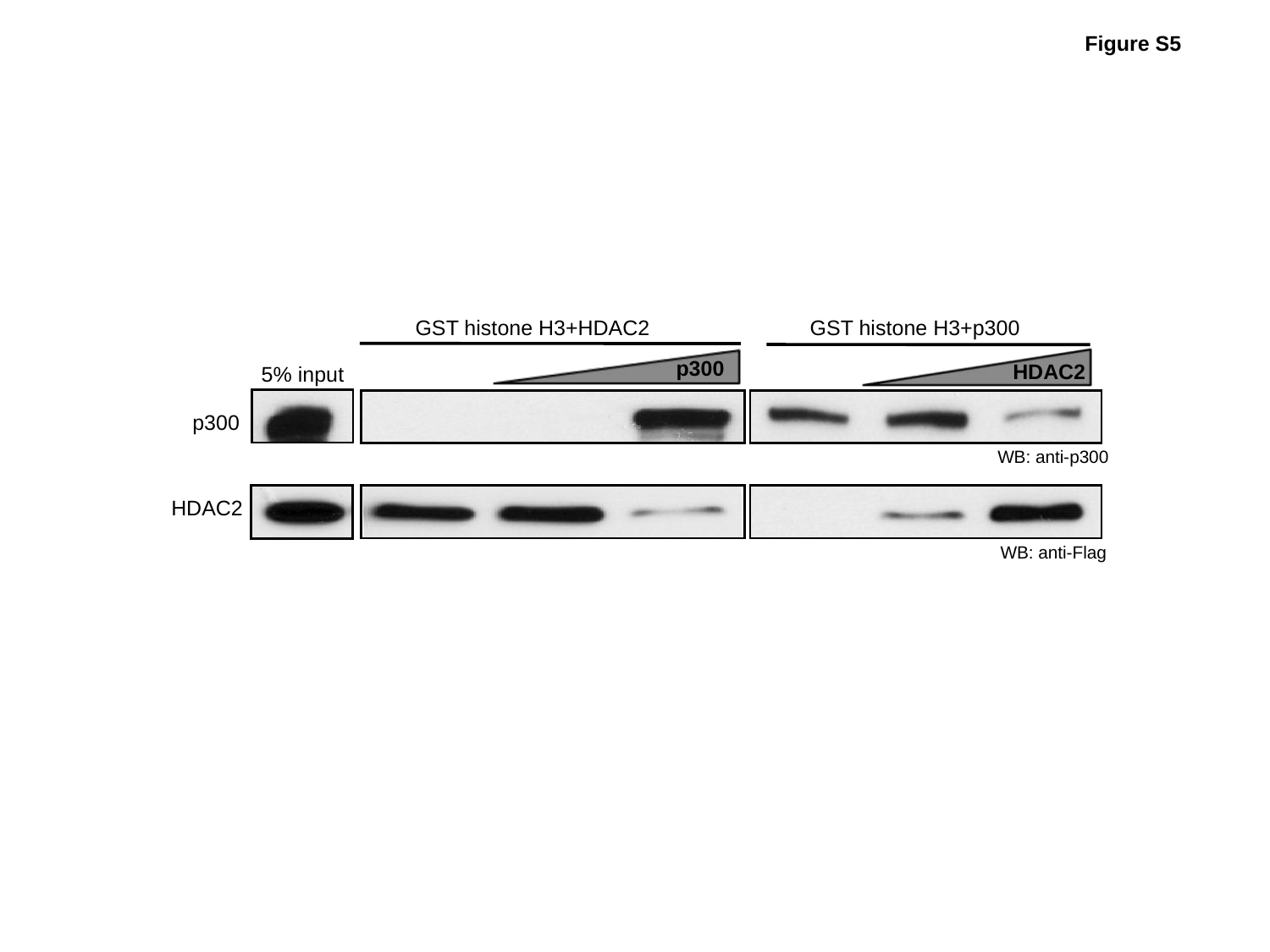

Figure S5
GST histone H3+HDAC2
GST histone H3+p300
p300
HDAC2
5% input
p300
HDAC2
WB: anti-p300
WB: anti-Flag

## Slide 6
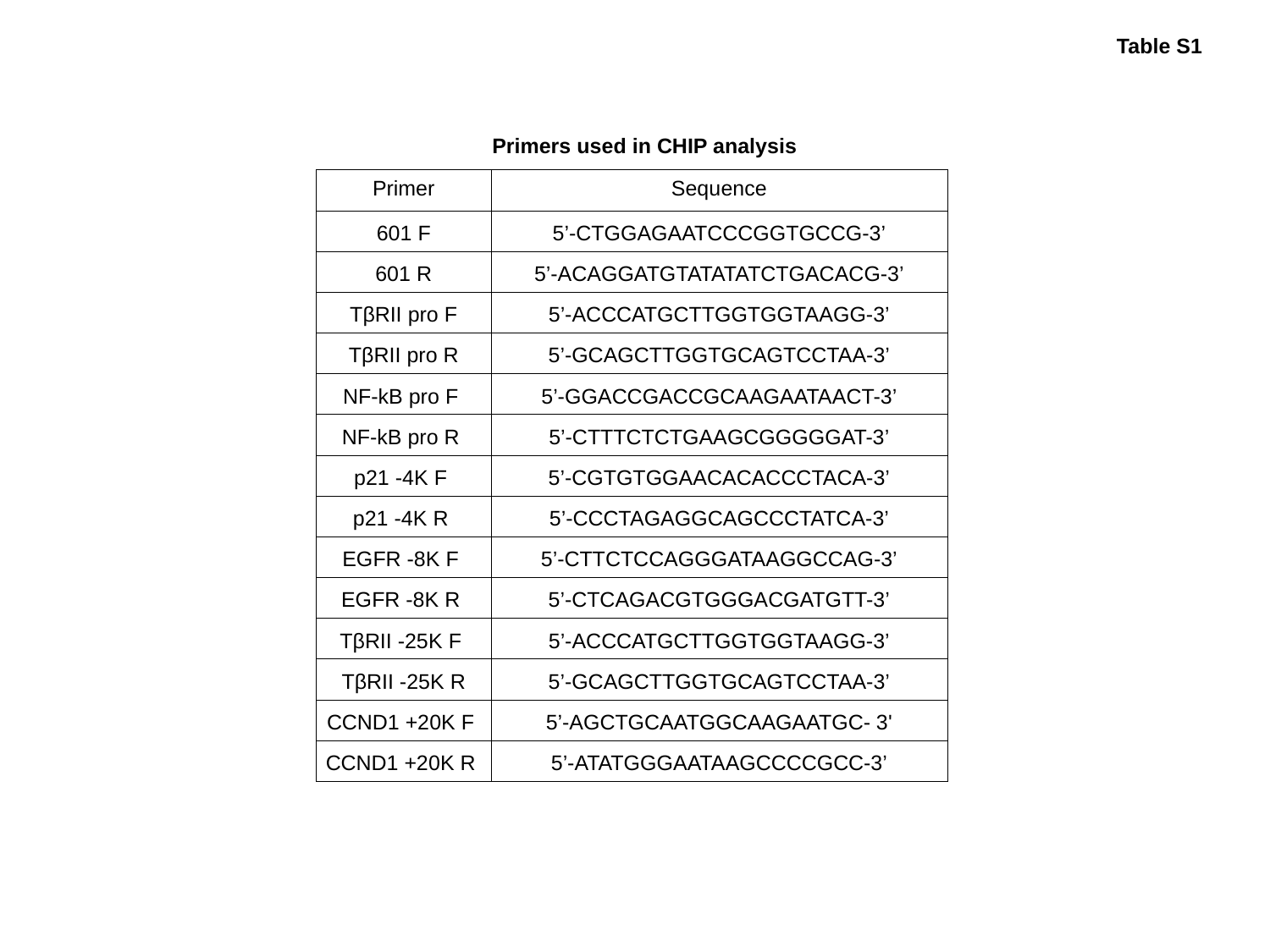

Table S1
Primers used in CHIP analysis
| Primer | Sequence |
| --- | --- |
| 601 F | 5’-CTGGAGAATCCCGGTGCCG-3’ |
| 601 R | 5’-ACAGGATGTATATATCTGACACG-3’ |
| TβRII pro F | 5’-ACCCATGCTTGGTGGTAAGG-3’ |
| TβRII pro R | 5’-GCAGCTTGGTGCAGTCCTAA-3’ |
| NF-kB pro F | 5’-GGACCGACCGCAAGAATAACT-3’ |
| NF-kB pro R | 5’-CTTTCTCTGAAGCGGGGGAT-3’ |
| p21 -4K F | 5’-CGTGTGGAACACACCCTACA-3’ |
| p21 -4K R | 5’-CCCTAGAGGCAGCCCTATCA-3’ |
| EGFR -8K F | 5’-CTTCTCCAGGGATAAGGCCAG-3’ |
| EGFR -8K R | 5’-CTCAGACGTGGGACGATGTT-3’ |
| TβRII -25K F | 5’-ACCCATGCTTGGTGGTAAGG-3’ |
| TβRII -25K R | 5’-GCAGCTTGGTGCAGTCCTAA-3’ |
| CCND1 +20K F | 5’-AGCTGCAATGGCAAGAATGC- 3' |
| CCND1 +20K R | 5’-ATATGGGAATAAGCCCCGCC-3’ |

## Slide 7
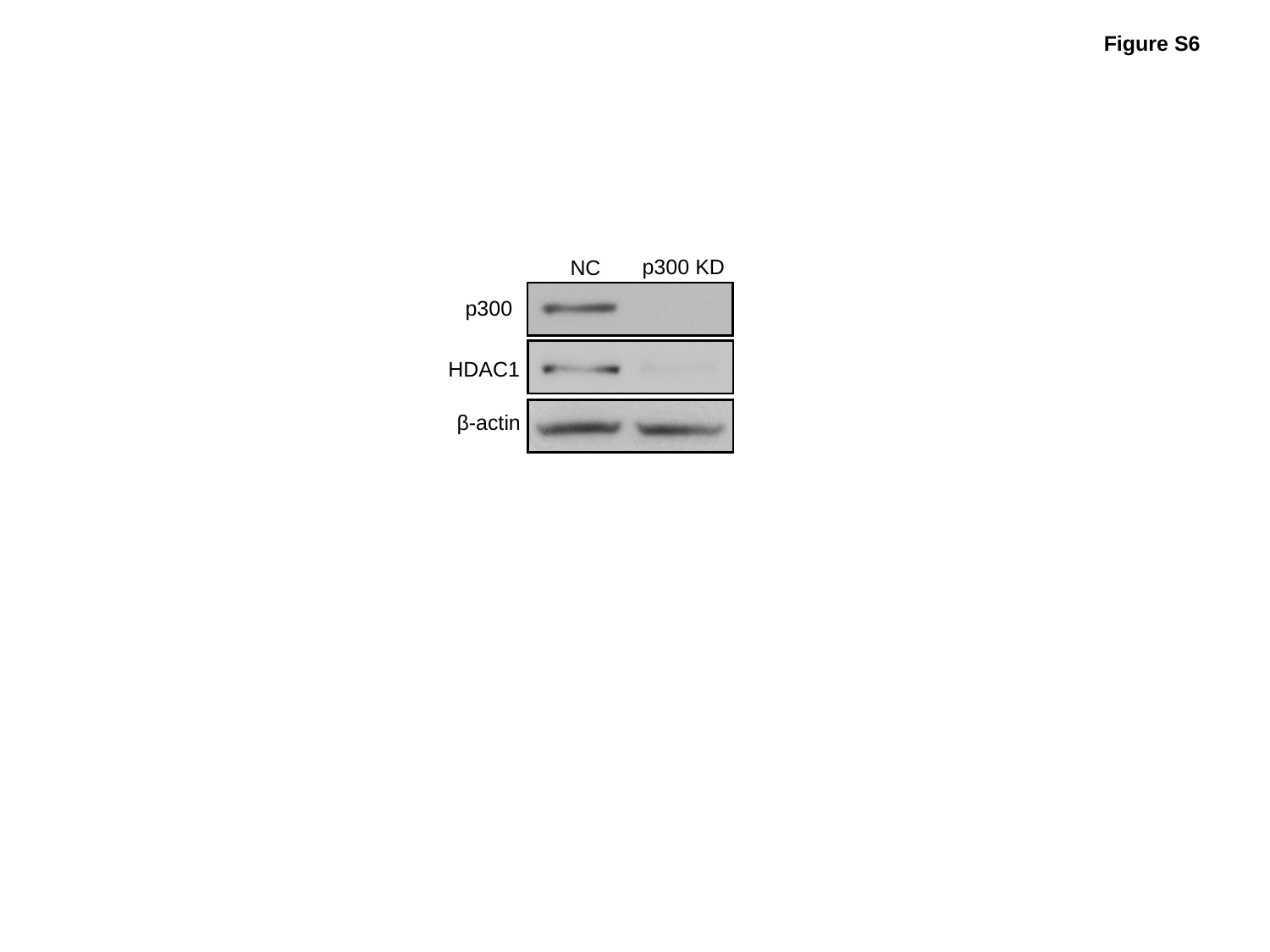

Figure S6
p300 KD
NC
p300
HDAC1
β-actin
